# Pyroptosis in Periprosthetic Osteolysis

**DOI:** 10.3390/biom12121733

**Published:** 2022-11-23

**Authors:** Jian Yin, Zhaoyang Yin, Peng Lai, Xinhui Liu, Jinzhong Ma

**Affiliations:** 1 Department of Orthopedics, Shanghai General Hospital of Nanjing Medical University, Shanghai 201600, China; 2 Department of Orthopedics, The Affiliated Jiangning Hospital with Nanjing Medical University, Nanjing 211100, China; 3 Department of Orthopedics, The Affiliated Lianyungang Hospital of Xuzhou Medical University (The First People’s Hospital of Lianyungang), Lianyungang 222000, China; 4 Department of Orthopedics, Shanghai General Hospital, Shanghai Jiao Tong University School of Medicine, Shanghai 201600, China

**Keywords:** periprosthetic osteolysis, aseptic loosening, NLRP3, pyroptosis, inflammation

## Abstract

Periprosthetic osteolysis (PPO) along with aseptic loosening (AL) caused by wear particles after artificial joint replacement is the key factor in surgical failure and subsequent revision surgery, however, the precise molecular mechanism underlying PPO remains unclear. Aseptic inflammation triggered by metal particles, resulting in the imbalance between bone formation by osteoblasts and bone resorption by osteoclasts may be the decisive factor. Pyroptosis is a new pro-inflammatory pattern of regulated cell death (RCD), mainly mediated by gasdermins (GSDMs) family, among which GSDMD is the best characterized. Recent evidence indicates that activation of NLRP3 inflammasomes and pyroptosis play a pivotal role in the pathological process of PPO. Here, we review the pathological process of PPO, the molecular mechanism of pyroptosis and the interventions to inhibit the inflammation and pyroptosis of different cells during the PPO. Conclusively, this review provides theoretical support for the search for new strategies and new targets for the treatment of PPO by inhibiting pyroptosis and inflammation.

## 1. Introduction

Total joint arthroplasty (TJA) is regarded as the most effective treatment for patients suffering from end-stage arthritis and provides satisfactory long-term stability for patients’ joint shape and function [1]. With the aging of the population and increment in traumatic accidents, the number of patients receiving TJA is dramatically increasing [2]. According to the statistics, approximately 1.5 million arthroplasties are performed worldwide each year. By 2030, joint replacement surgeries are projected to be 4 million per year in the United States [3,4]. However, despite ongoing improvements in prosthetic materials and surgical techniques, patients receiving TJA may be at risk for revision surgery, which brings a substantial public health burden worldwide [5,6]. Periprosthetic osteolysis (PPO) is a progressive and occult process of bone resorption after joint replacement [7]. Prior report shows aseptic loosening (AL) following PPO after TJA is one of the most common reason for surgical revision [8], accounting for 20.3% of cases in the USA [9]. The inflammatory response around the joint prosthesis, especially the activation of macrophages, plays a key role in the occurrence and development of PPO [10]. Gasdermins family-mediated pyroptosis is a pro-inflammatory regulatory cell death mode that involves the caspase cascade and the activation and release of pro-inflammatory factors, such as IL1β/18 [11,12]. Nevertheless, the role of the pyroptosis in PPO still remains controversial. Accordingly, we focus here, on the molecular mechanisms of pyroptosis in PPO, including its prime, activation, and downstream action, and the treatment strategy of PPO.

## 2. Pathogenesis of Periprosthetic Osteolysis

Bone is a metabolically active tissue composed of various types of cells. The homeostasis of bone metabolism is a complex biological process, including the differentiation of stem cells, the mineralization of osteoblasts and the phagocytosis of osteoclasts. Osteoclasts are derived from monocyte/macrophage precursors accompanied by overexpression of receptor activator of nuclear factor-κB ligand (RANKL) and RANK. Osteoblasts and osteoclasts are in a complementary and mutually regulated relationship. RANKL and intercellular adhesion molecule-1 (ICAM-1) expressed by osteoblasts can bind to RANK and lymphocyte function-associated antigen-1 (LFA-1) on the membrane surface of osteoclasts, respectively, which promote the activation of osteoclast precursors [13]. Conversely, osteoprotegerin (OPG), recognized as a negative regulator of osteoclastogenesis, is a decoy receptor for RANKL secreted by osteoblasts, which protects bone from osteolysis by binding to RANKL and preventing it from binding to RANK [14]. On the other hand, osteoclasts can regulate osteoblast function directly via intercellular interaction, or cytokines, such as semaphorin 4D, which is expressed by osteoclasts and binds to the receptor Plexin-B1 on osteoblasts to inhibit bone formation [15].

Osteolysis often occurs due to the disequilibrium between bone formation and bone resorption. Wear particles of different sizes and shapes generated at the areas of contact interface and impingement with the joint prosthesis after JTA operation are the initiator of PPO. Depending on the material of the prosthesis, wear particles can be classified into high-density polyethylene, tricalcium phosphate (TCP), bone cement as well as metal particles, including titanium (Ti), nickel (Ni), chromium (Cr) and cobalt (Co) [16,17,18,19] all of which are common inflammatory inducers in clinical practice [20]. Research indicates that factors that influence cellular responses to wear particles include particle composition, shape, size and charge [21]. Apparently, these inflammatory responses are mainly related to the phagocytosis of wear particles by macrophages/monocytes [22]. Micro/nano-scale wear particles activate a wide variety of periprosthetic cell types, such as osteoblasts, lymphocytes, fibroblasts, multinucleated foreign-body giant cells and macrophages [17,18,19,23], to secrete pro-inflammatory mediators along with chemotaxis factors, including tumor necrosis factor-alpha (TNF-α), macrophage colony-stimulating factor (M-CSF), interleukin (IL)-1β, IL-6, IL-17A [24], prostaglandin E2 (PGE2), vascular endothelial growth factor (VEGF) and RANKL [25,26,27] etc., which further drive osteoclast formation, differentiation and maturation, shifting bone metabolic homeostasis to osteolysis, ultimately leading to periprosthetic bone resorption and osteolysis [28,29]. AL secondary to PPO can be defined as a failure of the implant due to poor initial fixation and mechanical damage to fixation over time or biological loss of fixation instigated by immunological reactions to wear particles [30]. Accordingly, sterile inflammation caused by wear particle has been identified as the initiator of PPO and AL.

## 3. Pyroptosis in PPO

Cell death has been identified as necrosis, autophagy, apoptosis, ferroptosis, necroptosis and pyroptosis in mammalian cells. Since Friedlander first described anthrax lethal toxin causing macrophage death and rapid release of intracellular contents in 1986 [31], pyroptosis, a unique form of highly inflammatory regulated cell death (RCD), has gradually entered the field of view of researchers. In 2001, Cookson et al. first formally defined pyroptosis as a lytic, pro-inflammatory RCD characterized by caspase-1 activation; despite this, it was later shown to be an RCD that relies on the gasdermin protein as the executioner of cell death [32]. The committee on Cell Death Nomenclature (NCCD) defines pyroptosis in 2018 as a type of RCD that depends on gasdermin family proteins to form pores in the plasma membrane, usually but not always activated by caspases cascade, such as caspases-1/4/5/11 [33]. It is generally agreed that the classic mechanism of pyroptosis activation is inflammasome-caspase-1 pathway. Pathogen-associated molecular patterns (PAMPs) or damage-associated molecular patterns (DAMPs) activate the canonical inflammasome and promote the polymerization of apoptosis-associated speck-like protein containing a CARD (ASC). Inflammasome recruit caspase-1 through CARD-CARD interactions and cleave downstream substrates. As a cytosolic multimeric protein complex, an inflammasome is composed of sensor protein, caspase-1 family protease and the adaptor protein ASC. In addition to the cleavage of precursors of the inflammatory cytokines pro-IL-1β/18 into IL-1β/18, the activated caspase-1 substrates also triggers cleavage of Gasdermin D (GSDMD) into N-terminal fragment of GSDMD (GSDMD-NT) and C-terminal fragment of GSDMD (GSDMD-CT) [34]. The former inserts pores in the cell membrane and compromises membrane integrity, accompanied by the secretion of inflammatory signaling molecule, such as IL-1β/18 [35]. IL-1β is the most effective stimulator of macrophages by metal particles and is involved in the polarization and repolarization of M1 macrophages [36]. The extracellular IL-1β and IL-18 can induce a strong inflammatory response by activating the IL-1R/IL-18R-MyD88-NFκB pathway. Nonetheless, activation of IL-1β/18 is neither necessary nor sufficient for the occurrence of pyroptosis [37]. NF-κB is upstream of NLRP3 and activated NF-κB escalates the expression of inflammation-related genes NLRP3, IL-1β and IL-18 [38]. Additionally, non-canonical inflammasome directly senses cytosolic LPS from Gram-negative bacteria and their outer membrane vesicles through caspase-4/5 in humans or caspase-11 in mice. Similar to caspase-1, activated caspase-4/5/11 cleave GSDMD to induce pyroptosis, but cannot directly cleave pro-IL-1β/18 into IL-1β/18 [34,39]. An earlier study showed that caspase-3 and caspase-8 pro-enzymes were expressed in the interfacial membrane after aseptically loosened hip arthroplasty [40]. A recent finding unmasked that pathogenic *Yersinia* elicited receptor-interacting serine/threonine-protein kinase 1 (RIPK1)-dependent caspase-8 cleavage of gasdermin D (GSDMD) and pyroptosis by inhibiting macrophage transforming growth factor-β-activated kinase 1 (TAK1) [41]. Hauwermeiren F et al. revealed that *B. anthracis* infection triggers NLRP3 activation and caspase-8–dependent apoptosis of B6 macrophages [42]. However, to date, little is known about how this alternative caspase-8-dependent pyroptotic pathway is activated or regulated.

Activation of gasdermins (GSDMs), a family of porogens containing six gene clusters (GSDMA-E and pejvakin [PJVK]), is usually activated by the proteolytic removal of the auto-inhibitory carboxy-terminal domain of the caspase modulators [43], which is a necessary condition for the occurrence of pyroptosis [44]. GSDMB, GSDMC and GSDMD are only expressed in mammals [45]. GSDMD, a major member of GSDMs family, contains dual domain with autoinhibitory mechanism, whereby the GSDMD-CT possessing repressor activity constitutively interacts with and represses the GSDMD-NT possessing pore-forming activity [46]. The dual domain structure of GSDMD is linked by a domain containing a caspase-1/4/5 (in humans) or caspase-1/11 (in mice) cleavage site [34]. Upon cleavage by caspase-1/4/5/11, the autoinhibition of the C terminus is eliminated, releasing the p30 N terminal fragment with pore-forming activity (GSDMD-NT). Lipid binding triggers the oligomerization of GSDMD-NT to form transmembrane pores in the plasma membrane, leading to disruption of osmotic potential, swelling, terminal membrane rupture, as well as the release of mature IL-1β and IL-18 [35]. GSDMD-NT pore (approximately 18 nm inner diameter) provides a logical conduit through which IL-1β (17 kDa, 4.5 nm diameter) and IL-18 (18 kDa, 5.0 nm in diameter) escape the cell [47]. Intriguingly, PJVK contains only a small C-terminal part [45]. Strikingly, GSDMD pores can only allow small molecules to pass through, while large molecules, such as lactate dehydrogenase (LDH) (140 kDa), escape cells via plasma membrane rupture (PMR). PMR is a catastrophic event for pyroptosis. DAMPs released by PMR further amplify the inflammatory response, a biological process that can be regulated by the 16-kDa cell-surface Ninjurin (NINJ) 1 protein. NINJ1 is a novel adhesion molecule that is involved in many inflammatory diseases [48]. NINJ1 expression is ubiquitous, including in myeloid cells and the central nervous system (CNS) [49]. Recent studies have identified it as an important mediator of PMR in lytic cell death pathways, including pyroptosis, as well as an essential protein for pyroptosis-related PMR [50,51]. Although PMR, similar to GSDMD pore formation, is a pathological process of pyroptosis, most of the current studies have ignored this mechanism. Knockout of NINJ1 blocked macrophages to the post-stimulated PMR, resulting in the inability to release a variety of intracellular substances, including HMGB1 and LDH [51]. On the one hand, it limits the spread of the inflammation, and on the other hand, it also reduces the host’s anti-inflammatory ability. PMR has important implications for future studies of AL in PPO. 

The formation of GSDMs membrane pores is the hallmark event of pyroptosis, while inflammasome activation leads to IL-1β maturation and the initiation of pyroptosis [52]. The receptors in the inflammasome complex belong in most cases to the Nod-like receptor (NLR) and absent in melanoma (AIM)2-like receptor (ALR) families. NLRP1, NLRP3, NLRC4, NLRP6, NLRP7 and NLRP12 are members of the NLR family [53]. Among these inflammasomes, NLRP1, NLRP3, NLRP4, AIM2 and pyrin are the focus of current research [54]. AIM2 is a non-NLR inflammasome, which recognizes double-stranded DNA (dsDNA), either pathogen or host-derived, through its C terminal HIN200 domain. Binding of dsDNA to the HIN domain of AIM2 recruits ASC and procaspase-1, leading to the maturation of caspase-1 and pro-inflammatory cytokines [55]. Non-canonical inflammasomes include mouse caspase-11 and its highly homologous human caspase-4 and caspase-5. Although NLRP1 was the first inflammasome to be discovered [56], currently, NLRP3 is the mainstream inflammasome in PPO scientific research. During infectious bone resorption, LPS triggers an inflammatory cascade by activating NLRP3 inducing macrophages to release a series of pro-inflammatory cytokines, such as IL-1β, IL-6, and TNF-α, which subsequently accelerates bone destruction [57]. The NLRP3 inflammasome is composed by the NLRP3 receptor, ASC and caspase-1. The NLRP3 receptor consists of three components, namely the N-terminal pyrin domain (PYD), the central nucleotide-binding oligomer Domain (NACHT) and a C-terminal LRR domain. Once activated, NLRP3 oligomerizes via homotypic interactions of their NACHT domains. Oligomeric NLRP3 recruits ASC through homotypic PYD-PYD interaction and subsequently recruits caspase-1 via CARD-CARD interaction [54]. NLRP3 is mainly expressed in macrophages/microglia/osteoclasts, osteoblasts, dendritic cells, neutrophils and lymphocytes [58].

Recent evidence has confirmed that pyroptosis plays a critical role in various inflammation-related diseases such as myocarditis [59], neurodegeneration [60], or even cancer treatment [61]. However, whether pyroptosis contributes to wear particle-induced PPO warrants to be fully elucidated. Intriguingly, More and more evidences suggest that wear particles can accelerate PPO by activating NLRP3 [62,63]. Current evidences indicate that NLRP3 inflammasome can activate caspase-1/4/5 in humans or caspase-1/11 in mice [64,65]. In addition, cathepsin B (CTSB), a cysteine cathepsin associated with various diseases including rheumatoid arthritis, liver fibrosis and atherosclerosis [66,67], also has a pivotal role in the activation of NLRP3. In myeloid-derived suppressor cells, the chemotherapeutics gemcitabine and 5-fluorouracil depend on lysosomal permeabilization and release of CTSB, which binds to NLRP3 and drives caspase-1 activation, ultimately releases IL-1β and reduces anticancer immunity [68]. Chevriaux A et al. demonstrated that in BMDMs cells, CTSB interacts with NLRP3 at the endoplasmic reticulum (ER) level and leads to caspase-1 activation, IL-1β production, and ASC speck formation [69]. Endocytosis of crystalline or granular material by cells results in swelling and lysis of lysosomes and release of CTSB [70]. It is generally believed that wear particles are phagocytosed by circulating macrophages in the pathological process of osteoclastogenesis and bone loss (PPO). Due to the rupture of lysosomes, the protease CTSB leaks into the cytoplasm and activates the NLRP3 inflammasome, thereby inducing IL-1β maturation and secretion, and activating pyrolysis [71].

The biofilm formed between the prosthesis and the surrounding bone tissue contains a variety of cell types, such as osteoclasts, macrophages, osteoblasts, osteocytes, fibroblasts, and a small number of lymphocytes. To date, research on pyroptosis in PPO has focused on osteoclasts (macrophages), osteoblasts, and osteocytes [4,72]. In the following, we will review according to the classification of PPO-related pyroptosis of different cells.

## 4. Pyroptosis in Macrophage

Macrophages can be divided into M1-polarized pro-inflammatory phenotype and M2-polarized anti-inflammatory phenotype. The former can be activated by LPS and IFN-γ to aggravate the inflammatory response, while the latter can be activated by IL-1 and IL-13 to alleviate inflammation [73]. Osteoclasts are multinucleated giant bone-resorbing cells of the myeloid lineage, and their formation is a multistep process that requires macrophage colony-stimulating factor (M-CSF) and receptor activator of nuclear factor kappa B ligand (RANKL)/RANK activation [74]. Cathepsin K (CTSK), a critical specific lysosomal protease, is secreted by osteoclasts that contributes to bone matrix resorption [75]. In addition, osteoclast differentiation-related protein nuclear factor of activated T-cells (NFATc)-1 and extracellular matrix degradation-related protein matrix metalloprotein 9 (MMP9) are involved in the differentiation and maturation of osteoclasts.

**Figure 1:**
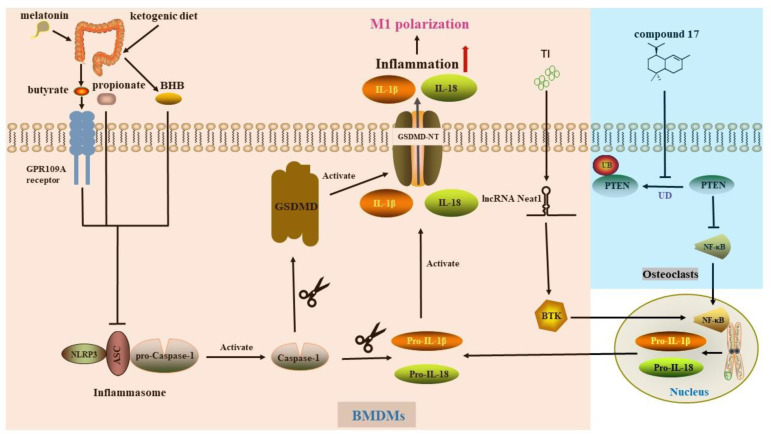
Pyroptosis in macrophages or osteoclasts. The NLRP3 inflammasome activates caspase-1, which activates GSDMD, the precursor of Pro-IL-1β and Pro-IL-18, respectively. Subsequent oligomerization of GSDMD-NT forms transmembrane pores in the plasma membrane, resulting in the release of IL-1β and IL-18. Melatonin increases butyrate content by affecting gut microbiota, which inhibits NLRP3 activation by binding to the GPR109A receptor. Both propionate and butyrate attenuate NLRP3 activation by inhibiting ASC assembly, but propionate is GPCRs-independent and HDAC-independent. BHB alleviates NLRP3 activation by inhibiting HDAC. TI particle stimulation up-regulates the expression levels of lncRNA Neat1 as well as BTK, aggravates the inflammatory response through the NF-κB pathway. Compound 17, a small molecule compound can rescue inflammatory osteolysis by inhibiting PTEN ubiquitination. PTEN inhibits the activation of NLRP3 and osteoclast activation by nuclear translocation of NF-κB p65. However, RANKL and LPS results in the ubiquitination of PTEN. BHB: β-hydroxybutyrate. BMDMs: bone marrow-derived macrophages. BTK: Bruton tyrosine kinase. Compound 17: a small molecule compound. GPCRs: G protein-coupled receptors. HDAC: histone deacetylase. lncRNA: long noncoding RNA. PTEN: phosphatase and tensin homolog. TI: titanium. UD: ubiquitination degradation.

A growing body of evidence has confirmed that inhibition of osteoclast activation is an effective strategy to hinder osteolysis. Regulation of inflammasome activation by a ketogenic diet (KD) has attracted increasing attention and has been applied in the research of various diseases, including Alzheimer’s disease [76,77]. The ketone body is composed of acetone, acetoacetate (AcAc) and β-hydroxybutyrate (BHB). Prior evidence has indicated that NLRP3 could be selectively blocked by concentrated extracellular K+, glyburide, MCC950, minocycline and KD [78,79]. The ketogenic diet (KD) refers to a low-carbohydrate and high-fat dietary strategy that was first used to treat intractable epilepsy by simulating a state of starvation [80]. The process of ketosis occurs in the mitochondria of cells, where acetyl-CoA from fatty acid catabolism is converted into ketone bodies, namely BHB, AcAc, and acetone [81]. Due to its significant efficacy in weight loss, KD has now emerged as a nutritional intervention for several metabolic disorders [82]. In addition, the ketogenic diet also has potential application value in the treatment of CNS diseases such as tumors, Parkinson’s syndrome, Alzheimer’s disease, and CNS injury [83,84]. Wu and his colleagues demonstrated that BHB, but not AcAc, inhibited CoCrMo particle-induced cleavage of caspase-1 precursor into the active form and activation of NLRP3 inflammasome in bone marrow-derived macrophages (BMDMs) by interfering with ASC assembly. This inhibitory effect of BHB on BMDMs was independent of G protein-coupled receptor (GPR)109a and histone deacetylase (HDAC), whereas the inhibitory effect on osteoclast differentiation was mainly dependent on HDAC. BHB inhibited osteoclast differentiation in vitro by reducing the expression of TRAF6 and NFATc-1, meanwhile, decreased the levels of MMP9, CTSK and tartrate-resistant acid phosphatase (TRAP) to hinder the bone resorption function. Nutritional intervention with BHB similarly blocked the differentiation and function of osteoclasts in mice undergoing osteolytic surgery. In summary, BHB, including a KD, may alleviate CoCrMo alloy particle-induced osteolysis via inhibiting the NLRP3-GSDMD inflammatory pathway [85]. Another experiment by the same research group showed that oral administration of melatonin affected the relative abundance of certain short-chain fatty acid (SCFA)-producing bacteria in the gut as well as the amount of butyrate instead of acetate and propionate, in a Ti particle-induced mice calvarial osteolysis model. Subsequently, they were further confirmed in GPR109A knockout mice that butyrate reduced the expression levels of NLPR3 along with caspase-1 and alleviated osteolysis through binding to the GPR109A receptor. Strikingly, the pyroptosis-dependent protein GSDMD was also not detected [63]. Another study by the same researching group unmasked that propionate and butyrate inhibited NLRP3 activation by hindering ASC oligomerization and speckle formation in BMDMs cells, although the effect of propionate was independent of GPRs or HDAC inhibitors, butyrate required GPR109A. In addition, propionate and butyrate reduced the IL-1β-induced elevation of osteoclast differentiation-related proteins, including TRAF2, TRAF6, NFATc-1 and c-Fos, by inhibiting HDAC, and ultimately blocked the formation of osteoclasts [86]. Relieving inflammation by modulating gut microbiota and diet structure provides a novel potential therapeutic target for the treatment of PPO (Figure 1).

Calcitonin receptor (CTR) is a cell surface receptor that affects osteoclast-mediated bone resorption [87]. Studies have found that melatonin-induced calcitonin can inhibit osteoclast activity [88]. Dendritic cell-specific transmembrane protein (DC-STAMP) is a key molecule mediating osteoclast fusion [89]. Accumulating evidence has shown that NFATc-1 is a major transcriptional regulator of osteoclast differentiation and regulates the expression of TRAP, CTSK, DC-STAMP and CTR in osteoclast [90]. Deletion of NFATc-1 results in blocked osteoclast formation. Disruption of NFATc-1 in hematopoietic cells has been reported to contribute to a decrease in osteoclast formation and an increase in bone mass [91]. Upon binding of RANK to the ligand RANKL on osteoclasts, the RANK/tumor necrosis factor receptor-associated factor 6 (TRAF6)/TAK1 complex phosphorylates IκB kinases (IKKs), subsequently activates the NF-κB signaling cascade. Specifically, activated IKKs promote the degradation of IκB and relieve its inhibitory effect on NF-κB. Afterwards, NF-κB p65 is translocated to the nucleus, facilitating the transcription of NFATc-1 and the differentiation of osteoclasts [92,93].

NF-κB p65 nuclear translocation is a canonical pathway for LPS-induced NLRP3 activation [94], a process that is restrained by phosphatase and tensin homolog (PTEN), a well-known tumor suppressor [95,96]. In previous studies, various methods, such as microRNA and catalpol, inhibited the RANKL-induced NF-κB signaling pathway or PI3K/Akt pathway downstream of PTEN, thereby inhibiting the proliferation and differentiation of osteoclasts [97,98,99]. Interestingly, PTEN affects NF-κB signaling pathway during ANKL-induced osteoclastogenesis [100,101]. Homologous to E6AP C terminus (HECT) domain ligases transfer ubiquitin to PTEN and induce its ubiquitination [102]. Y Chen and colleagues [103] prepared a small-molecule compound named compound 17 structurally based on heclin, a HECT ligase inhibitor, and found that this compound could inhibit the ubiquitination of PTEN in osteoclasts (derived from BMDMs) stimulated by RANKL and LPS, thereby inhibiting IκBα and NF-κB p65 phosphorylation and NF-κB nuclear translocation. Decreased release of NLRP3 and IL-1β restrained osteoclast activation and bone resorption capacity, which was protective against inflammatory osteolysis. Moreover, VO-Ohpic, a specific inhibitor of PTEN, counteracted this protective effect. Taken together, reducing the activation of the downstream NLRP3 inflammasome and osteoclast activation by inhibiting the ubiquitination of PTEN may be an effective way to rescue inflammatory osteolysis.

Bruton tyrosine kinase (BTK), a potent pro-inflammatory factor, has been shown to promote the oligomerization of ASC and the activation of caspase-1 [104]. Lin et al. discovered that BTK knockout reduced the phosphorylation of IKK β along with p65, increased the level of I κB α, and alleviated the inflammation of BMDMs induced by Ti particles by inhibiting the NF-κB pathway. Upstream of BTK, long noncoding RNA (LncRNA) Neat1 positively regulated BTK to promote inflammation through two sets of pathways, namely impeding the KLF4 expression to inhibit its inhibitory effect on BTK transcription, as well as acting as a miRNA sponge for miR-188-5p to promote BTK translation. Stimulation of Ti particles increased the expression level of lncRNA Neat1 in BMDMs. The authors applied si-Neat1 to interfere with its expression, which significantly inhibited the NF-κB pathway and NLRP3 activation, and finally reversed osteolysis (Figure 1). Neat1 has the potential to be one of the promising targets for preventing AL [105]. Moreover, some scholars used adipocyte exosomes to encapsulate miR-34a to inhibit Ti-induced polarization of M1-type macrophages by inhibiting NLRP3 activation [106]. 

## 5. Pyroptosis in Osteocyte

**Figure 2:**
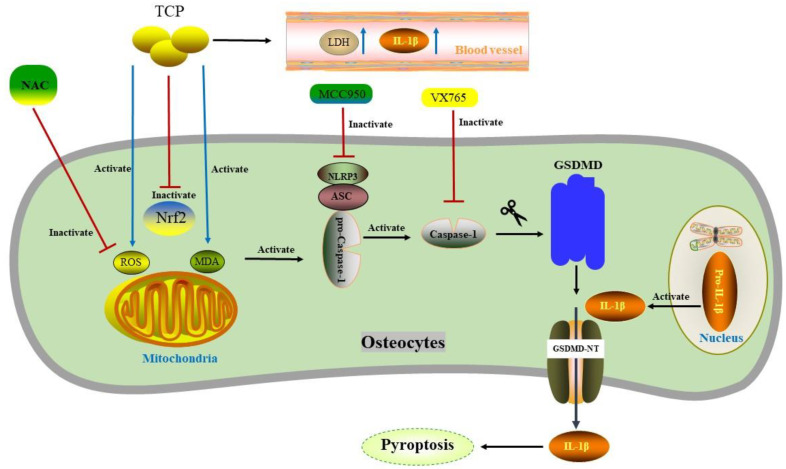
TCP-mediated pyroptosis of calvarial osteocytes. TCP up-regulates the levels of ROS and MDA in osteocytes, as well as the activation of NLRP3-mediated osteocytes pyroptosis. In addition, the concentrations of IL-1β and LDH in serum increases. These effects can be reversed by the caspase-1 inhibitor VX765 or the NLRP3 inhibitor MCC950 or the ROS scavenger NAC. TCP: tricalcium phosphate. ROS: reactive oxygen species. MDA: malonaldehyde. Nrf2: antioxidant enzyme nuclear factor E2-related factor 2. LDH: lactate dehydrogenase. NAC: N-acetylcysteine.

Osteocytes are terminally differentiated cells, accounting for more than 95% of all cells in bone. Osteocytes are involved in bone growth, bone modeling and bone remodeling [107]. A model of calvarial osteolysis induced by tricalcium phosphate (TCP) demonstrated that TCP particles significantly up-regulated the ratio of Caspase-1 p20/Caspase-1, IL-1β/pro-IL-1β, and the expression levels of NLRP3 and GSDMD-N [108]. Additionally, serum IL-1β and LDH concentrations were also elevated. Interestingly, these effects could be reversed by the caspase-1 inhibitor VX765 or the NLRP3 inhibitor MCC950. Reactive oxygen species (ROS) is one of the critical factors that activates the NLRP3 inflammasome [16]. TCP particles significantly increased ROS and malonaldehyde (MDA) generation in calvarial osteocytes, whereas it restrained the expression of antioxidant enzyme nuclear factor E2-related factor 2 (Nrf2). Application of the ROS scavenger N-acetylcysteine (NAC) can significantly inhibit the activation of NLRP3 and osteocytes pyroptosis. By this token, ROS may mediate TCP-osteocyte pyroptosis and accelerate periprosthetic osteolysis [108] (Figure 2). In other recent studies, the application of N-[2-bromo-4-(phenylsulfonyl)-3-thienyl]-2-chlorobenzamide, Astragalin, and Alpinetin all alleviated inflammatory osteolysis by reducing ROS production [109,110,111]. Consequently, ROS scavengers will be a new potential approach for the treatment of osteoclast-related inflammatory osteolytic diseases.

## 6. Pyroptosis in Osteoblast

Osteoblasts derived from bone marrow mesenchymal stem cells are the main source of bone cells. The balance between osteoblasts and osteoclasts maintains the homeostasis of bone metabolism [13]. Sirtuin3 (SIRT3), a well-studied mitochondrial nicotinamide adenine dinucleotide (NAD) +-dependent deacetylase of mitochondria, exerts direct effects on inflammasome assembly and activation. In macrophages, SIRT3 activates the NLRC4 inflammasome through deacetylation to help clear infection. However, for the activation of NLRP3 and AIM2, SIRT3 is not necessary [112]. However, in a study conducted by K Zheng et al. [113] on Ti-induced osteolysis of rat femurs, it was found that depletion of SIRT3 enhanced the activation of the NLRP3. The researchers also confirmed that the expression of SIRT3 in the revision TJA was significantly lower than that in the primary TJA through the investigation of clinical samples. Targeted upregulation of SIRT3 significantly restrained Ti particles-induced activation of NLRP3 and the expression levels of pyroptosis-related proteins, including caspase-1, GSDMD, IL-1β and IL-18. Meanwhile, the periprosthetic bone mineral density, trabeculae and bone mass were preserved. Intriguingly, the anti-osteolytic effect of SIRT3 was reversed after the application of indocyanine green-001, a targeted Wnt/β-catenin inhibitor. Collectively, SIRT3 inhibits NLRP3 inflammasome activation and pyroptosis dependent on Wnt/β-catenin signaling pathway, and alleviates Ti particles-induced osteolysis by promoting osteogenesis, which makes modulation of SIRT3 a potential treatment for PPO [113]. Furthermore, the treatment strategies mentioned above to regulate inflammation and pyroptosis for the treatment of PPO and AL were summarized in Table 1.

## 7. Discussion

PPO and AL have become one of the most common long-term complications after total joint arthroplasty. Biological and mechanical factors, including the prosthesis material, the method of surgery, and infection within the joint are contributing factors [114,115]. Strikingly, sterile inflammation in macrophages and osteoclasts triggered by wear debris, such as metal particles is the leading cause. Apart from metal particles, ultra-high molecular weight polyethylene particles can also stimulate bone cells to secrete prostaglandin E2 to activate osteoclasts [116]. With the advancement of materials science, ceramic materials with good tissue compatibility and low wear rate are gradually used in clinical practice. In clinical follow-up, ceramic prosthesis achieved better clinical efficacy [117]. There are few reports on the experimental research of ceramics in PPO. Osteoimmunology is involved in various physiological and pathological processes of bone, including the activation of the inflammasome, the release of inflammatory factors and the revitalization of inflammatory cells. 

Pyroptosis is an important pro-inflammatory RCD with important roles in macrophage M1-type polarization and osteoclast activation. As touched upon earlier, the interface membrane between the joint prosthesis and the surrounding bone tissue contains multiple cell types, such as macrophages, osteoblasts, osteoclasts, and fibroblasts. At present, research concerning pyroptosis in PPO are mainly concentrated in osteoclasts. Scholars have alleviated osteolysis by using various drugs and measures to inhibit wear particle-induced pyroptosis of macrophages, osteoblasts and osteocytes, as well as activation of osteoclasts. As the number of fibroblasts accounts for more than 70%, the contribution of fibroblasts to bone loss during PPO remains enigmatic [7,118,119]. It is worth noting that fibroblasts are also an important source of inflammatory stimuli such as RANKL, which deserves further study. In the process of PPO, in addition to the enhancement of osteoclastogenesis, the inhibition of osteogenic activity is also an important etiology. Although some scholars have focused on the interaction between different types of cells [120,121], further research is still needed. 

The formation of pores in GSDMs is the key to the occurrence of pyroptosis. At present, the research on pyroptosis mainly focuses on GSDMD. Other GSDMs, such as GSDME, may also plays an active role in PPO. In addition, although NLRP3 and caspase-1 have received a lot of attention, few studies to date concentrate on other inflammasomes or caspase cascade, such as AIM2 and caspase-8. While our understanding of the mechanisms of pyroptosis is rapidly improving, drugs and interventions that can effectively inhibit pyroptosis and alleviate PPO in clinical practice are still in their infancy. Strategies to suppress pyroptosis include ROS scavenger (e.g., Prussian blue nanozyme) [122], caspase-1 inhibitors (e.g., VX-765, VX-740 and Z-YVAD-FMK) [123,124,125], NLRP3 inhibitors (e.g., MCC950, CY09 and OLT1177) [126,127,128] and GSDMD inhibitors (e.g., disulfiram, Ac-FLTD-CMK) [129,130]. Although scholars have used a variety of drugs and methods, such as exosomes, lncRNAs, chemical agents, and KDs, etc. to alleviate metal particle-induced pyroptosis and achieved meaningful experimental conclusions; many compelling questions remain for future investigation.

Recent studies suggest that the absence of caspase-8 hindered the activation of caspase-1 and caspase-11 by NLRP3 [131], which is a potential target for pyroptosis regulation. Additionally, GSDMD silencing may be a promising strategy in the future. The crosstalk between different types of RCD, such as autophagy, apoptosis, ferroptosis, and pyroptosis etc., needs to be fully investigated. Collectively, further work is still needed to elucidate the role of pyroptosis in PPO, and interventions to inhibit pyroptosis and inflammation may serve as rational new treatments against PPO and AL.

## Figures and Tables

**Table 1:** Research on inflammasomes and osteolysis.

Therapeutic Target/Agents	Animal	Cell Type	Animal Model	In Vitro Cell Model	Caspase	Gasdermins	Inflammasome	Ref.
BHB	C57BL/6J mice (male)	BMDMs	Calvarial osteolysis induced by CoCrMo alloy particles (average 1.69 μm)	Stimulated by LPS and CoCrMo alloy particles	Caspase-1	GSDMD	NLRP3	[63]
Melatonin	C57BL/6J mice (male)	BMDMs	Calvarial osteolysis induced by Ti particles (average 500 nm)	Stimulated by LPS and Ti particles	Caspase-1	Not detected	NLRP3	[52]
propionate, butyrate	C57BL/6J mice (male)	BMDMs	Calvarial osteolysis induced by CoCrMo alloy particles	Stimulated by LPS and CoCrMo alloy particles	Caspase-1	GSDMD	NLRP3	[64]
compound 17 small molecule	C57BL/6J mice (female)	Osteoclasts induced from BMDMs	Calvarial osteolysis induced by LPS	Osteoclast stimulated by RANKL or LPS	Caspase-1	Not detected	NLRP3	[78]
Neat1 downregulation (si-Neat1)	C57BL/6J mice (male)	BMDMs	Calvarial osteolysis induced by Ti particles	Stimulated by LPS and Ti particles	Caspase-1	Not detected	NLRP3	[80]
miR-34a carried by EXOs	C57BL/6J mice (male)	RAW 264.7	Calvarial osteolysis induced by Ti particles	Stimulated by Ti particles	Not detected	Not detected	NLRP3	[81]
NAC VX765 * MCC950 **	ICR mice (male)	Osteocytes	Calvarial osteolysis induced by TCP particles (average 1.997 μm)	Not established	Caspase-1	GSDMD	NLRP3	[82]
SIRT3	SD rat (male)	MSCs and Osteoblast	Femoral osteolysis induced intramedullary implantation of Ti rod and particles	MSCs stimulated by Ti particles	Caspase-1	GSDMD	NLRP3	[84]

* Caspase-1 inhibitor; ** NLRP3 inhibitor. Abbreviations (in alphabetical order): BHB: ketone body β-hydroxybutyrate; BMDMs: bone marrow derived macrophages; EXOs: exosomes; LPS: lipopolysaccharide; NAC: N-acetylcysteine (a reactive oxygen species scavenger); TCP: tricalcium phosphate; Ti: titanium; MSCs: mesenchymal stem cells.
